# Editorial: Deep learning for neurological disorders in children

**DOI:** 10.3389/fncom.2022.984882

**Published:** 2022-08-04

**Authors:** Saman Sargolzaei

**Affiliations:** Department of Engineering, University of Tennessee at Martin, Martin, TN, United States

**Keywords:** children, deep learning, neurological disorder, artificial intelligence, machine learning, fMRI, EEG, neuroimaging

## Aims and objectives

Children are among the vulnerable age population to the incidents and consequences of various neurological disorders. The vulnerability extends beyond the long-lasting health and socioeconomic impact on the patients and families by introducing unique challenges in studying pediatric neurological disorders. Among the distinct challenges are age-specific technological requirements design considerations and the inter-woven nature of such studies' findings with downstream mechanisms and manifestations related to child growth and development.

Deep learning (DL), a subdiscipline of machine learning and artificial intelligence (AI) in a broader context, has provided a unique prospect to unravel complexities in diverse applications, including medicine and biology. The emergence of DL-based approaches in studying pediatric neurological disorders while relying on high-dimensional data and computing capacity could lead to a better understanding of downstream pathways, more accurate diagnosis, and more efficient management plans.

The Research Topic, entitled *Deep learning for neurological disorders in children*, aimed at conveying scientific studies and discoveries related to the role of DL in studying pediatric neurological disorders. Among the main themes called for the topic were (1) prospects of DL frameworks in diagnosis and prognosis of pediatric neurological disorders and delineating downstream pathways of such disorders and (2) DL-driven opportunities to overcome the challenges associated with studying neurological disorders in children. The Research Topic concluded with eight original research articles (in the order of their publication date, Nemzer et al.; McNorgan; Da Silva et al.; Abdelhameed and Bayoumi; Almuqhim and Saeed; Attallah; Hosseini et al.; Molina-Maza et al.), one brief research report article (Laria et al.) and one review article (Sargolzaei et al.). The following section briefly summarizes these contributions and corresponding developments. However, readers are encouraged to consider the summary as a starting point and refer to each contribution to learn more.

## Contributions and developments

Most of the contributions were centered around classifying data between healthy controls and disorders and identifying within disorders classes and disorders-driven events. Targeted disorders included attention deficit hyperactivity disorders (Laria et al.), autism spectrum disorders (Almuqhim and Saeed; Hosseini et al.), dyslexia (McNorgan; Da Silva et al.), epilepsy (Abdelhameed and Bayoumi), and childhood medulloblastoma (Attallah). The developed DL-based classifiers utilized various data modalities, including behavioral assessment (Laria et al.), electroencephalography (EEG) (Abdelhameed and Bayoumi), functional magnetic resonance imaging (fMRI) (McNorgan; Da Silva et al.; (Almuqhim and Saeed), histopathological imaging (Attallah), and facial imaging (Hosseini et al.).

Another interesting main facet of the contributions was the DL utilization for developing simulated neuronal models to aid a better understanding of epileptic seizures (Nemzer et al.) and automatic resolution enhancement of pediatric brain magnetic resonance imaging acquired in downsized time (Molina-Maza et al.). These developments can be viewed as potential pathways DL can overcome technical challenges entangled with studying pediatric neurological disorders (Sargolzaei et al.).

As for data sources, contributions made use of a variety of dataset repositories and initiatives, such as OpenNeuro (reported in McNorgan), BraIns initiative (reported in Da Silva et al.), PhysioNet (reported in Abdelhameed and Bayoumi), ABIDE (reported in Almuqhim and Saeed), IEEE DataPort (reported in Attallah), and Kaggle (reported in Hosseini et al.).

## Perspectives and insights

Considering the contributions, Sargolzaei et al. can be regarded as a possible entry gateway supplying the reader with history and a scoping review of DL's role in the current field of focus before the presented topic. Furthermore, the contributions exemplify a wide range of DL solutions in terms of theoretical and methodological advancements and enabling decision aids in diagnosing pediatric neurological disorders.

Nemzer et al. discussed Hodgkin-Huxley simulations on small-world network topology to comprehend neuronal behavior linked to epileptic seizures. Such developments will ease fine-tuned research for specific neurological conditions and expand access to the collection of synthetic data for training purposes. Molina-Maza et al. developed a DL-based scheme for automatic resolution enhancement of pediatric brain MRI acquired in downsized time, aiming to advance free-sedation pediatric brain imaging protocols.

Tying the DL realm with classical statistical models is an exciting domain of investigation. Laria et al. introduced dlasso, a neural network version of the lasso algorithm for studying ADHD symptom severity. Regarding probing neural mechanisms and decision aids in learning abilities, McNorgan employed DL joined with brain functional connectivity network to examine reading-skill-dependent mechanisms and their impact on cognitive domains.

Access to domain-specific tools that interpret DL models can lead to a more informed understanding. Da Silva et al. developed a tool called VisualExpplanations, to classify developmental dyslexia while unraveling brain mechanisms insights facilitating the DL models findings interpretation in conjunction with neuroscientific knowledge.

The power of DL solutions is optimized when the structure and pipeline are carefully planned. Abdelhameed and Bayoumi proposed a DL approach for the task of automatic EEG-based seizure detection in pediatric patients while emphasizing the significance of structure fine-tuning and attention to data elements. Attallah developed the CoMB-Deep pipeline to classify childhood medulloblastoma and identify tumor sub-classes based on histopathological images.

Timely diagnosis of pediatric neurological disorders necessitates using all existing data modalities. Almuqhim and Saeed developed ASD-SAENet for the task of ASD classification using fMRI data. Autism classification was also the focus of Hosseini et al., where a MobileNet-based DL model was developed using facial image analysis.

## Remarks and recommendations

While the route forward is exhilarating for applying DL models in studying pediatric neurological disorders, it shouldn't be conceived as a magic crystal ball. Instead, the more sharing practices we implement in collected data and developed models, the higher the chances are for realizing DL as one clinically approved solution that outperforms traditional diagnostic and prognostic solutions.

Among the relatively untouched areas of investigation are DL frameworks enabling the fusion of multi-modalities and taking advantage of basic science experimentations to guide the structure and implementation of DL models in studying neurological disorders in children.

## Author contributions

All authors listed have made a substantial, direct, and intellectual contribution to the work and approved it for publication.

## Conflict of interest

The author declares that the research was conducted in the absence of any commercial or financial relationships that could be construed as a potential conflict of interest.

## Publisher's note

All claims expressed in this article are solely those of the authors and do not necessarily represent those of their affiliated organizations, or those of the publisher, the editors and the reviewers. Any product that may be evaluated in this article, or claim that may be made by its manufacturer, is not guaranteed or endorsed by the publisher.

